# Do weaker solvation effects mean better performance of electrolytes for lithium metal batteries?†

**DOI:** 10.1039/d5sc01495f

**Published:** 2025-03-31

**Authors:** Liang Li, Kaixiang Ren, Wenjun Xie, Qi Yu, Shilin Wu, Hai-Wen Li, Meng Yao, Zhipeng Jiang, Yongtao Li

**Affiliations:** a School of Materials Science and Engineering, Anhui University of Technology Maanshan 243002 China jzp1994@ahut.edu.cn liyongtao@ahut.edu.cn; b Key Laboratory of Efficient Conversion and Solid-State Storage of Hydrogen & Electricity of Anhui Province Maanshan 243002 China; c School of Advanced Energy, Sun Yat-sen University Shenzhen 518107 China; d College of Materials Science and Engineering, Sichuan University 610065 Chengdu Sichuan People's Republic of China

## Abstract

The design of novel electrolytes is crucial for ensuring the practical application of high-voltage lithium metal batteries (LMBs). Weakly solvating electrolytes (WSEs), achieved by reducing the solvent's solvation ability, have been shown to improve the cycling stability of LMBs. However, the internal relationship between the weaker solvation effects of the solvent and battery performance is not well understood. In this work, we design a series of solvents with different solvation effects, using 1,2-dimethoxyethane (DME) and 1,3-dioxolane (DOL) as base skeletons, and systematically examine the physicochemical and electrochemical properties of these WSEs. Our results show that the performance of WSEs is not solely determined by the solvation structure but is also influenced by the intrinsic reactivity of the solvent. Guided by this principle, we develop a high-performance electrolyte based on 2-methoxy-1,3-dioxolane (2-MeO DOL), which exhibits both weak solvation effects and low reactivity. This electrolyte enables stable cycling of Li–Cu half cells for over 250 cycles with a coulombic efficiency of 99.3% and demonstrates stable cycling in Li–LiNi_0.8_Co_0.1_Mn_0.1_O_2_ (NCM811) full cells at a 4.4 V cut-off voltage under practical conditions. This study offers critical insights for the design and high-throughput screening of next-generation high-performance WSEs for LMBs.

## Abstract 2

The electrochemical performance of weakly solvating electrolytes for lithium metal batteries depends not only on the solvation properties but also on the reactivity of the solvent.

## Graphical abstract

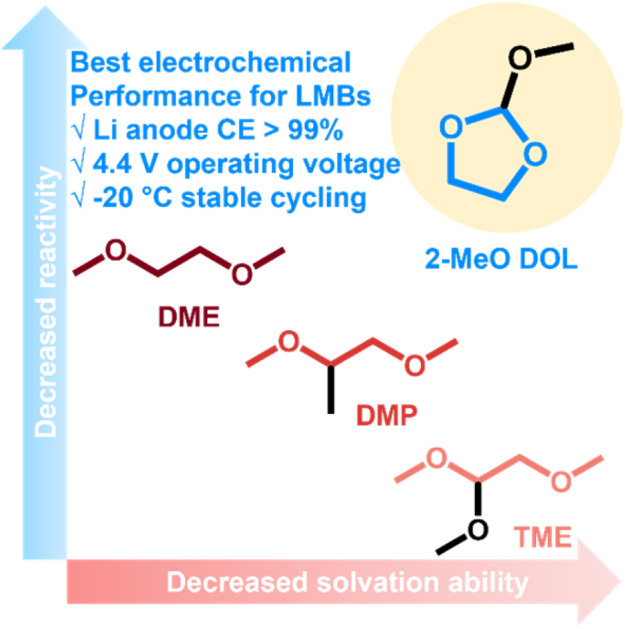

## Introduction

Lithium metal batteries (LMBs) are considered one of the most promising energy storage systems for the future, offering exceptional energy densities due to the high theoretical specific capacity (3860 mA h g^−1^) and low reduction potential (−3.04 V *versus* the standard hydrogen electrode) of metallic Li.^1,2^ However, the practical application of LMBs is currently limited by several challenges, including short cycle life and safety concerns.^3,4^ The lithium metal anode (LMA) faces issues such as dendritic growth, an unstable solid electrolyte interphase (SEI), and poor Li deposition/stripping efficiency.^5,6^ Additionally, high-energy-density LMBs with high-voltage cathodes (*e.g.*, LiNi_0.8_Co_0.1_Mn_0.1_O_2_ (NCM811), LiCoO_2_ (LCO)) are plagued by an unstable cathode-electrolyte interphase (CEI), structural degradation, and active material loss.^7,8^ Electrolyte engineering is an effective strategy for addressing these issues, as the electrolyte interacts with both the anode and cathode.^9^ Tuning its composition can simultaneously improve both interfaces, leading to enhanced cycling stability for LMBs.^10^

Conventional liquid electrolytes typically consist of Li salts, solvents, additives, and co-solvents (or diluents). Much of the research in electrolyte engineering focuses on modifying these components to optimize electrochemical performance. Liu *et al.* synthesized a novel Li salt, lithium (trifluoromethanesulfinyl)(trifluoromethanesulfonyl)imide (LiSTFSI), which decomposes to form a bilayer interface that improves the lifespan of LMBs.^11^ Wang *et al.* introduced 1,2-diethoxypropane (DEP) as a fluoride-free solvent that broadens the electrochemical window, improving compatibility with high-voltage cathodes.^12^ Yan *et al.* developed Li_2_ZrF_6_ as a new electrolyte additive that forms an SEI rich in t-Li_2_ZrF_6_*in situ*, enhancing ionic conductivity and suppressing dendrite growth.^13^ Jiang *et al.* employed fluorobenzene (FB) as a cost-effective co-solvent to develop high-performance dilute high-concentration electrolytes (DHCEs) for practical LMBs.^14^

The solvation structure of the electrolyte plays a critical role in determining its properties, both in bulk and at the interface.^15^ Strongly solvating solvents such as 1,2-dimethoxyethane (DME), 1,3-dioxolane (DOL) and ethylene carbonate (EC) tend to cause Li salts to dissociate fully, resulting in solvation structures enriched in solvent-separated ion pairs (SSIPs).^16^ Although these structures facilitate high ionic conductivity, the large number of solvent molecules in the solvation shell is adverse to anion decomposition at the interphase, creating an unstable interfacial layer. Recent studies suggest that reducing the solvation ability of solvents can alter the solvation structure, resulting in weakly solvating electrolytes (WSEs) that promote the formation of contact ion pairs (CIPs) and aggregates (AGGs).^17–21^ This enhances anion decomposition and stabilizes the SEI, thereby improving the cycling lifespan and high-voltage stability of LMBs. For example, the Wang group demonstrated that a tetrahydropyran (THP)-based weakly solvating electrolyte enables stable cycling of LMBs at a cutoff voltage of 4.5 V.^22^ Bao *et al.* synthesized fluorinated 1,4-dimethoxylbutane (FDMB), a novel weakly solvating solvent, which shows excellent stability on both the Li anode and high-voltage cathodes.^23^ Coskun *et al.* synthesized 1,1,1-trifluoro-2,3-dimethoxypropane (TFDMP), which increases the oxidation stability of the electrolyte to 4.8 V and prevents LiFSI-induced corrosion of aluminum foil.^24^ While many studies attribute improvements in cycling performance to the formation of an inner solvation sheath rich in anions resulting from WSEs, the question remains: do weaker solvation effects necessarily lead to better performance of electrolytes for LMBs?

**Fig. 1:**
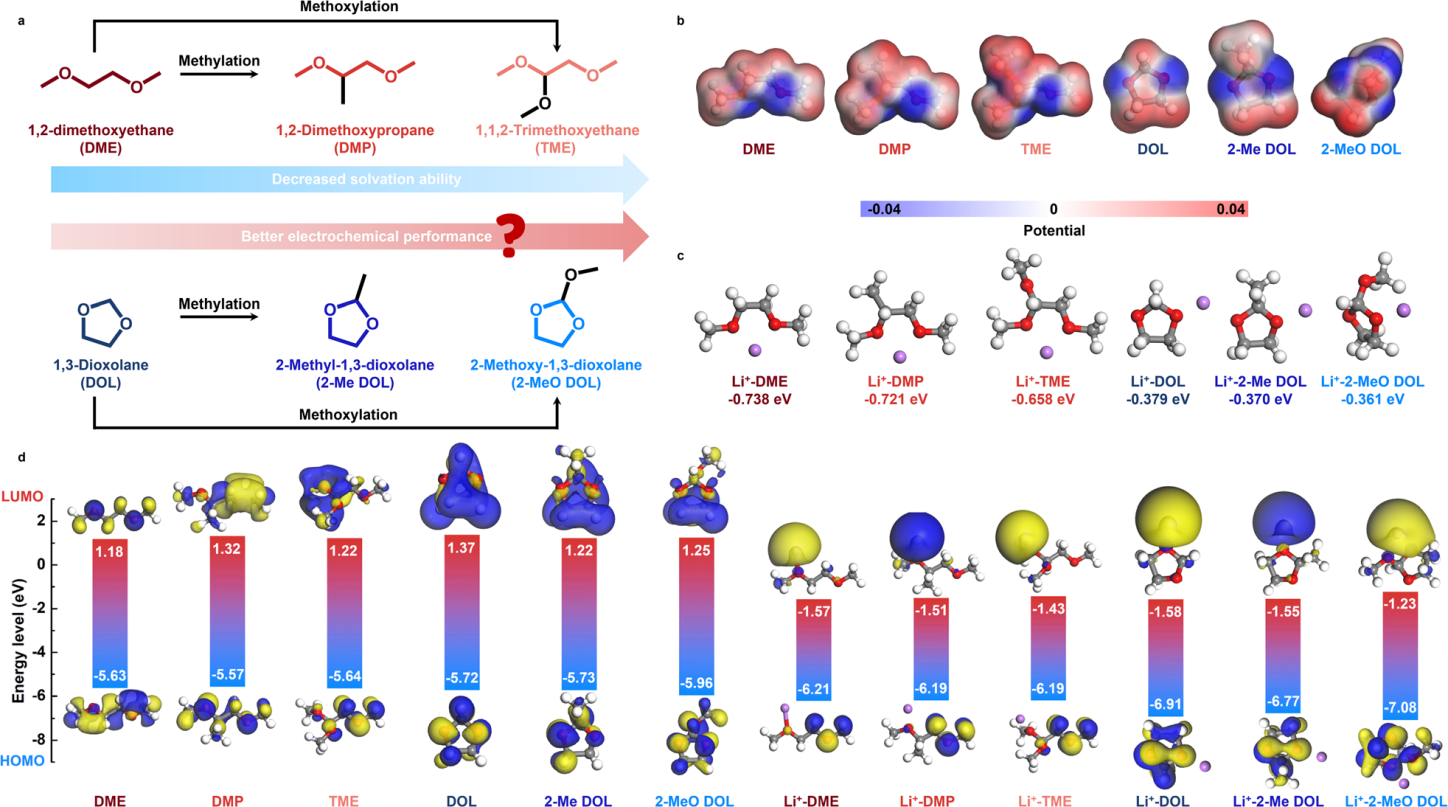
(a) Schematic diagram of the molecular design of weakly solvated chain ethers and cyclic ethers. (b) ESP maps of various ether solvent molecules. (c) Bonding energy of Li^+^ with different ether solvent molecules. (d) HOMO–LUMO levels of different solvent molecules and aggregates bound to Li^+^.

In this study, we investigate a series of solvents with varying solvation abilities, systematically examining their solvation structures, interfacial chemistry, and electrochemical performance. As shown in Fig. 1a, we selected typical linear ether DME and cyclic ether DOL as base skeletons and introduced methyl and methoxy groups into their structures, resulting in various WSEs. While significant progress has been made in developing methylated/methoxylated DOL-based WSEs, prior research has primarily focused on elucidating solvation structures, often overlooking the intrinsic chemical reactivity of solvent species.^25^ Here, we systematically examine both solvation structure and solvent stability, assessing their combined influence on the electrochemical performance of LMBs. Our results reveal that the electrochemical performance of electrolytes is not solely determined by the strength of the solvation effect but is also influenced by the inherent reactivity of the solvent. Ultimately, the 2-methoxy-1,3-dioxolane (2-MeO DOL)-based electrolyte, which balances weak solvation effects with low reactivity, exhibited optimal stability for both the LMA and high-voltage cathodes. This electrolyte enabled Li–Cu half cells to cycle stably for over 250 cycles with an average coulombic efficiency (CE) of 99.3%. Furthermore, it enabled Li-NCM811 cells to cycle stably at a cutoff voltage of 4.4 V, even under low-temperature (−20 °C) and practical test conditions (50 μm Li and 14.0 mg cm^−2^). This work provides essential insights into the design of high-performance WSEs.

## Results and discussion

We selected DME and DOL as the base solvent frameworks and introduced methyl and methoxy groups into their molecular structures. This led to the creation of four individual solvents: 1,2-dimethoxypropane (DMP), 1,1,2-trimethoxyethane (TME), 2-methyl-1,3-dioxolane (2-Me DOL), and 2-MeO DOL. Notably, no additional heteroatoms, such as F or N, were incorporated into the solvent molecules to avoid potential decomposition reactions that could lead to the formation of inorganic species like LiF or Li_3_N, which could confound experimental results.^26^ We employed density functional theory (DFT) to investigate the electrostatic potential (ESP) distribution maps of these solvents and to calculate the binding energies of each solvent with Li^+^. DFT results reveal that the methyl group induces steric hindrance, reducing the ability of adjacent oxygen atoms to coordinate with Li^+^ (Fig. 1b and c).^27^ Additionally, the electron-donating effect of the methyl group diminishes the electron density around the O atom, leading to a lower binding energy to Li^+^. Specifically, the binding energies of DME, DMP, DOL, and 2-Me DOL with Li^+^ are −0.738 eV, −0.721 eV, −0.379 eV, and −0.370 eV, respectively. Linear ethers exhibit higher binding energies than cyclic ethers, likely due to the more favorable chelation effect of linear ethers with Li^+^, resulting in stronger solvation ability.^28^ On the other hand, the methoxy group, due to its larger steric hindrance and stronger electron-donating conjugative effects, significantly weakens the O atom's coordination, resulting in lower solvation ability.^29^ The interaction energies between Li^+^ and TME and Li^+^ and 2-MeO DOL are −0.658 eV and −0.361 eV, respectively. Additionally, the highest occupied molecular orbital (HOMO) and lowest unoccupied molecular orbital (LUMO) energy levels of the free solvents and their Li^+^-bound forms were computed, providing insight into their oxidation and reduction reactivity (Fig. 1d). The HOMO energy levels of free DMP (−5.57 eV) and bound DMP (−6.19 eV) are higher than those of DME (−5.63 eV and −6.21 eV, respectively), suggesting that DMP is more prone to oxidation and exhibits higher reactivity. Similarly, the HOMO energy level of bound TME (−6.19 eV) is also higher than that of bound DME. In contrast, 2-MeO DOL shows lower HOMO energy levels, both in its free (−5.96 eV) and bound states (−7.08 eV), indicating superior oxidative stability. Furthermore, the LUMO energy level of bound 2-MeO DOL (−1.23 eV) is the highest among all bound ethers, suggesting its excellent reduction stability. The above theoretical calculation results show that 2-MeO DOL exhibits the weakest solvation ability and the lowest reactivity.

**Fig. 2:**
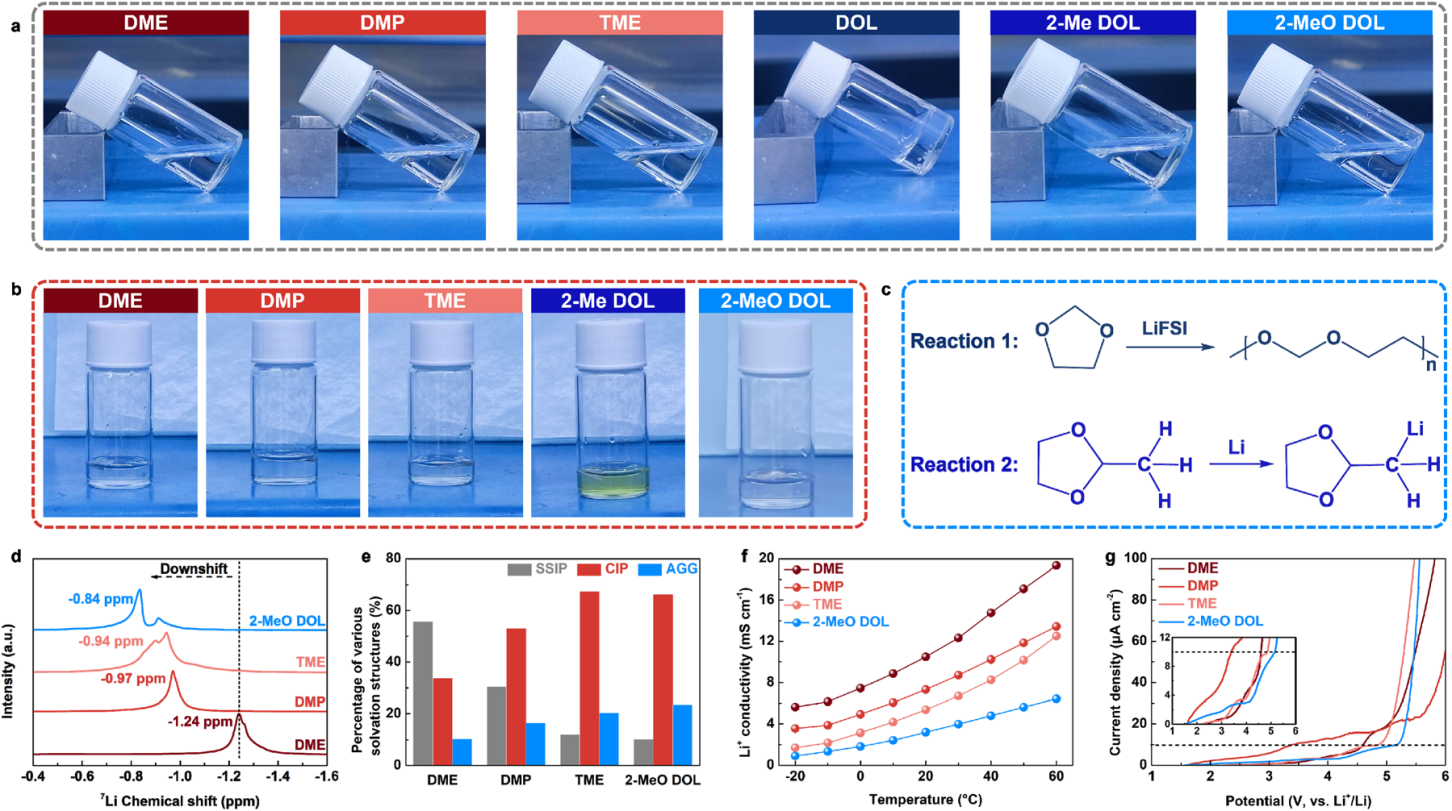
(a) Photos of electrolytes prepared by dissolving 1.5 M LiFSI in different ether solvents. (b) Photos of Li foil placed in different ether electrolytes for 3 h. (c) Possible reaction mechanism of DOL and 2-Me DOL. (d) ^7^Li NMR spectra, (e) solvation structure distribution, (f) Li^+^ conductivity, and (g) LSV plots of different ether electrolytes.

We dissolved 1.5 M lithium bis(fluorosulfonyl)imide (LiFSI) in the aforementioned solvents to prepare a series of electrolytes, which were subsequently analyzed for their physicochemical properties and solvation structures. As illustrated in Fig. 2a, after standing for 3 h, the DOL-based electrolyte exhibited noticeable polymerization, while the other electrolytes remained in the liquid state. This polymerization is attributed to the ring-opening polymerization of DOL induced by LiFSI.^30,31^ The introduction of substituents increases steric hindrance, thereby restricting the ring-opening polymerization of the cyclic ether.^32^ Furthermore, when metallic Li was immersed into the five electrolytes that remained stable in the liquid form, the 2-Me DOL-based electrolyte changed color from transparent to pale yellow, suggesting that 2-Me DOL is unstable in the presence of metallic Li and undergoes a chemical reaction (Fig. 2b).^33^ The mechanism of this reaction is illustrated in Fig. 2c. Based on these results, DME, DMP, TME, and 2-MeO DOL were selected for further study due to their stability with both the Li salt and metallic Li. We then recorded the ^7^Li nuclear magnetic resonance (NMR) spectra of the four electrolytes, revealing chemical shifts of −1.24 ppm, −0.97 ppm, −0.94 ppm, and −0.84 ppm (Fig. 2d). These downshift trends indicate a weakening of solvation ability in the following order: DME > DMP > TME> 2-MeO DOL, which is consistent with previous computational results.^34^ Raman spectra were deconvoluted to determine the solvation structure distributions (Fig. 2e and S1†).^35^ As the solvation ability weakened, SSIP decreased while AGG increased, suggesting the formation of an anion-rich inner solvation shell in WSEs (Fig. S2–S5†). Molecular dynamics (MD) simulations were performed to gain deeper insights into the solvation structure of 2-MeO DOL (Fig. S6†). The simulations reveal that FSI^−^ anions are the primary coordinating species in the first solvation shell of Li^+^ (Fig. S7†), as evidenced by radial distribution functions showing their dense spatial distribution around Li^+^ (Fig. S8†). These computational results corroborate the anion-rich solvation structure inferred from Raman spectroscopic analysis. These findings were further corroborated by ionic conductivity measurements, which revealed that, at varying temperatures, the ionic conductivities followed the trend: DME > DMP > TME > 2-MeO DOL, indicating that 2-MeO DOL exhibits the weakest solvation capacity (Fig. 2f). To assess the high-voltage stability of these electrolytes, we conducted linear sweep voltammetry (LSV) tests. Notably, the LSV results did not align directly with the solvation structure distribution (Fig. 2g). Despite DMP's weaker solvation, its oxidative stability was poorer than that of DME, with decomposition occurring below 3.5 V. This behavior is consistent with energy level analysis, which suggests that DMP, with a higher HOMO energy level, is more susceptible to oxidation. In contrast, 2-MeO DOL demonstrated the best oxidative stability, with a stable voltage window extending beyond 5 V. This enhanced stability is attributed to both the weak solvation structure formed by 2-MeO DOL and its low reactivity.

**Fig. 3:**
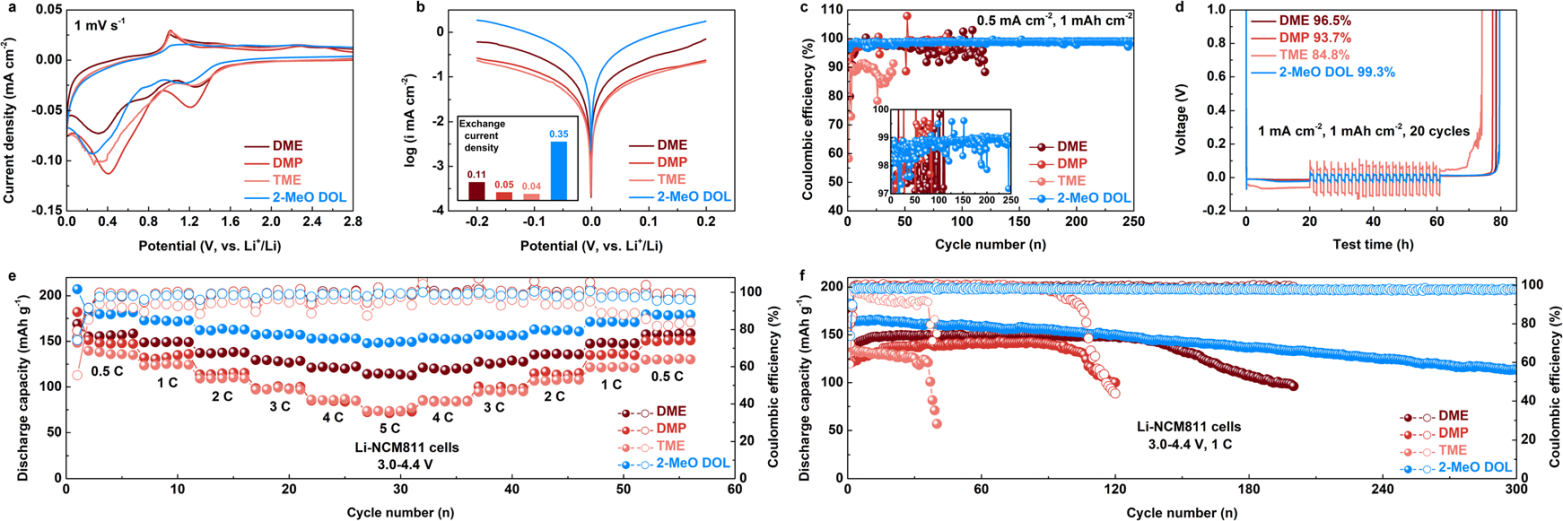
(a) CV curves of Li–Cu half cells using different ether electrolytes (scan rate: 1 mV s^−1^). (b) Tafel plots of Li–Li symmetrical cells using different ether electrolytes (scan rate: 1 mV s^−1^). (c) Cycling performance of Li–Cu half cells with various ether electrolytes (0.5 mA cm^−2^ and 1 mA h cm^−2^). (d) CE test of Li–Cu half cells using different electrolytes (1 mA cm^−2^, 1 mA h cm^−2^, and 20 cycles). (e) Rate performance, and (f) long-term cycling performance comparison of Li-NCM811 cells assembled with different ether electrolytes (3.0–4.4 V, 1C for long-term cycling test, and 1C = 180 mA g^−1^).

Cyclic voltammetry (CV) test results indicate that all electrolytes exhibit distinct peaks corresponding to the decomposition of the Li salt and solvent at approximately 1.3 V and 0.4 V (Fig. 3a).^36^ Notably, the intensity of the solvent decomposition peak during the reduction process is significantly higher in DMP- and TME-based electrolytes compared to DME and 2-MeO DOL electrolytes. This implies that, although an anion-rich inner solvation sheath forms in DMP and TME electrolytes, the solvents' high reactivity results in unavoidable side reactions with the Li anode during reduction. Differential pulse voltammetry (DPV) measurements demonstrate that DMP and TME produce a significantly enhanced current response around 0.3 V relative to other electrolytes (Fig. S9†).^37^ This pronounced reduction peak suggests that these components are more prone to electrochemical decomposition, leading to the formation of unstable organic byproducts. These results are in agreement with CV data, further supporting the impact of electrolyte composition on interfacial stability. Tafel analysis reveals that the exchange current densities of the SEI formed after cycling in DME, DMP, TME, and 2-MeO DOL electrolytes are 0.11, 0.05, 0.04, and 0.35 mA cm^−2^, respectively (Fig. 3b). These data indicate that Li^+^ deposition kinetics are fastest in the 2-MeO DOL-based electrolyte, whereas the kinetics in DMP and TME electrolytes are slower, even underperforming relative to DME.^38^ These differences in kinetic behavior strongly influence the cycling stability of the Li anode. As shown in Fig. 3c, under cycling conditions of 0.5 mA cm^−2^ and 1 mA h cm^−2^, the DME-based electrolyte supports stable cycling for 100 cycles, albeit with significant fluctuations in CE. In contrast, DMP- and TME-based electrolytes show stable cycling for fewer than 50 cycles, with TME demonstrating much lower CE per cycle. The Li–Cu half-cell assembled with the 2-MeO DOL-based electrolyte cycles stably for over 250 cycles, maintaining a high CE of nearly 99% per cycle. We used Adams's methodology to precisely quantify the CE of these electrolytes at the Li anode (Fig. 3d).^39^ After 20 cycles under 1 mA cm^−2^ and 1 mA h cm^−2^ conditions, the average CEs of DME, DMP, TME, and 2-MeO DOL electrolytes were found to be 96.5%, 93.7%, 84.8%, and 99.3%, respectively. Furthermore, the Li-NCM811 cell assembled with the 2-MeO DOL-based electrolyte demonstrated superior rate performance and cycling stability. With a cutoff voltage of 4.4 V, the battery delivered a discharge capacity of 148.7 mA h g^−1^ at 5C and demonstrated stable cycling for over 300 cycles at 1C. By contrast, the DME-based electrolyte exhibited a discharge capacity of 114.6 mA h g^−1^ at 5C, with significant capacity loss after more than 150 cycles at 1C (Fig. 3e, f and S10–S17†). Despite its lower ionic conductivity, the 2-MeO DOL-based electrolyte enables Li-NCM811 cells to achieve reduced interfacial impedance (*E*_sei_) in initial-cycle electrochemical impedance spectroscopy (EIS) measurements (Fig. S18†). This is primarily due to its anion-rich solvation structure, which drives the formation of an inorganic-rich SEI layer. Additionally, the electrolyte's high stability limits the formation of organic components, leading to a more robust and passivating interfacial layer that enhances long-term electrochemical performance. Notably, despite the presence of a high concentration of CIP and AGG in DMP and TME-based electrolytes, these systems perform worse than the DME-based electrolyte, which contains substantial SSIP. These results confirm that electrolyte performance is not solely dependent on the solvation structure but also strongly influenced by the intrinsic reactivity of the solvent.

**Fig. 4:**
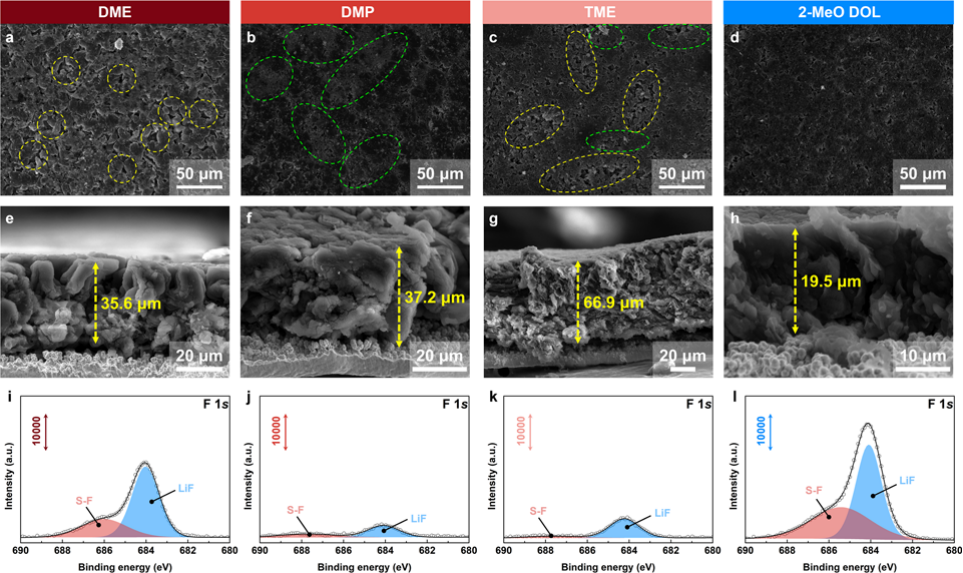
(a–d) Top-view and (e–h) side-view SEM images of metallic Li deposited in different ether electrolytes. The yellow frame represents the hole and the green frame represents the side reaction product. (i–l) XPS F 1s spectra of Li deposits obtained with various electrolytes. (a, e and i) 1.5 M LiFSI in DME. (b, f and j) 1.5 M LiFSI in DMP. (c, g and k) 1.5 M LiFSI in TME. (d, h and l) 1.5 M LiFSI in 2-MeO DOL (deposition conditions: 0.5 mA cm^−2^ for 3 mA h cm^−2^).

We further investigated the effects of different electrolytes on Li deposition morphology by depositing metallic Li with the same areal capacity (3 mA h cm^−2^). Scanning electron microscopy (SEM) images revealed that Li deposited from the DME-based electrolyte exhibited a porous structure, with a deposition thickness of 35.6 μm, corresponding to a 137% volume expansion relative to the theoretical thickness (15 μm) of Li (Fig. 4a and e).^40,41^ In the DMP-based electrolyte, the surface of the deposited Li showed no obvious pores but exhibited a significant presence of side reaction products, with a deposition thickness of 37.2 μm and a volume expansion of 148% (Fig. 4b and f). In TME-based electrolytes, the deposited Li exhibited both a porous morphology and side reaction products, with a thickness of 66.9 μm and a volume expansion rate of 346% (Fig. 4c and g). In contrast, Li deposited from the 2-MeO DOL electrolyte exhibited a compact structure with no significant porosity or side products (Fig. 4d). The deposition thickness was 19.5 μm, corresponding to a volume expansion rate of 30% (Fig. 4h). These observations explain the superior electrochemical performance of the 2-MeO DOL-based electrolyte, while TME demonstrates the poorest performance. X-ray photoelectron spectroscopy (XPS) analysis revealed that, although an anion-rich inner solvation sheath forms in DMP and TME electrolytes, the high reactivity of these solvents leads to significant incorporation of side reaction products into the SEI, resulting in a C/F ratio notably higher than that in DME and 2-MeO DOL electrolytes (Fig. S19†).^42^ Furthermore, the LiF content in the F 1s spectra is lower in DMP and TME (Fig. 4i–l). In contrast, the SEI derived from 2-MeO DOL exhibited the lowest C/F ratio (5.01) and a higher LiF content, suggesting that 2-MeO DOL, through weak solvation effect, promotes anion decomposition while maintaining solvent stability, forming an SEI enriched in LiF, which offers enhanced protection to the Li anode (Fig. S20–S23†).

**Fig. 5:**
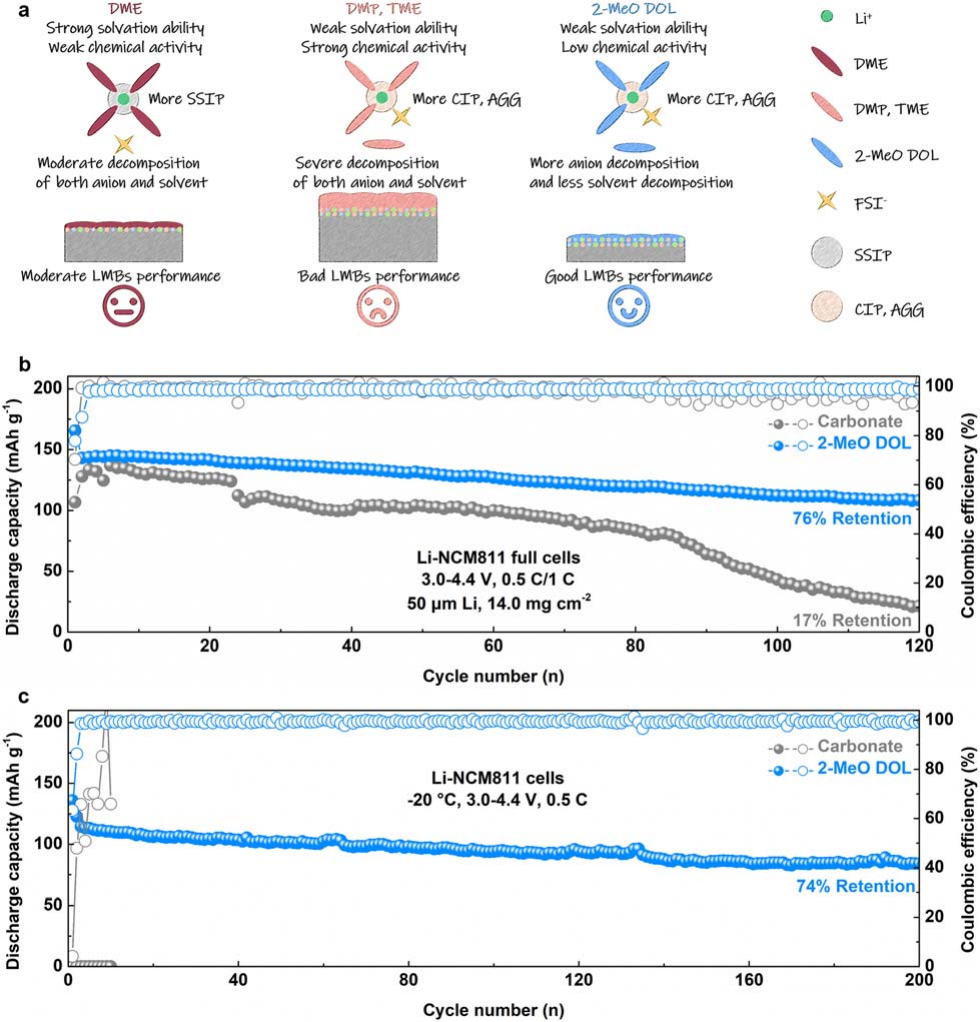
(a) Schematic diagram of the solvation structure of different ether electrolytes and their effects on LMA. (b) Long-term cycling performance of Li-NCM811 full cells using 1.5 M LiFSI in 2-MeO DOL and carbonate electrolytes (14.0 mg cm^−2^, 50 μm Li, 3.0–4.4 V, and 0.5C/1C). (c) Low-temperature cycling performance of Li-NCM811 cells using 1.5 M LiFSI in 2-MeO DOL and carbonate electrolytes (−20 °C, 3.0–4.4 V, and 0.5C).

We propose the following design principles for high-performance WSEs, as shown in Fig. 5a. While DME possesses a strong solvation ability and forms a significant number of SSIP structures, its low reactivity results in simultaneous moderate anion and solvent decomposition at the electrode–electrolyte interface, which limits its ability to improve the performance of LMBs. DMP and TME are weakly solvating solvents that promote the formation of CIP and AGG structures. However, their high intrinsic reactivity leads to substantial anion and solvent decomposition at the interface, resulting in the formation of SEIs rich in organic components, which hinders stable cycling performance. In contrast, 2-MeO DOL, with its combination of weak solvating ability and low reactivity, promotes anion decomposition while inhibiting solvent decomposition. This balance facilitates the formation of an SEI that is rich in inorganic components, significantly enhancing the performance of LMBs. To verify this conclusion, we assembled a practical Li-NCM811 full cell utilizing the 2-MeO DOL-based electrolyte under practical conditions (50 μm Li and 14.0 mg cm^−2^). Under cycling conditions of 0.5C/1C, the conventional carbonate electrolyte (1 M LiPF_6_ in EC/DEC) showed only 17% capacity retention after 120 cycles. In contrast, the Li-NCM811 full cell using the 2-MeO DOL-based electrolyte retained 76% of its initial capacity after the same number of cycles (Fig. 5b, S24 and S25†). Post-mortem analysis of these batteries showed that the X-ray diffraction (XRD) pattern intensity of the NCM811 cathode cycled with the 2-MeO DOL electrolyte was higher than that of the cathode cycled with the carbonate electrolyte, suggesting a lower level of side reaction products (Fig. S26†).^43^ Moreover, inductively coupled plasma mass spectrometry (ICP-MS) analysis of the cycled Li anode confirmed that the anion-rich CEI formed by 2-MeO DOL effectively mitigates active material loss (Fig. S27†).^44^ The F 1s spectrum of the cathode obtained by XPS analysis showed higher LiF content in the 2-MeO DOL electrolyte compared to the carbonate electrolyte, confirming its superior ability to promote anion decomposition (Fig. S28 and S29†). Additionally, 2-MeO DOL maintains good conductivity at low temperatures, enabling stable cycling of the Li-NCM811 cell at −20 °C for over 200 cycles at 0.5C, with 74% capacity retention, while the carbonate electrolyte, which freezes under similar conditions, renders the Li-NCM811 cell failure (Fig. 5c and S30†).^45^ This suggests that the weakly solvated and low-reactive 2-MeO DOL-based electrolyte can enable stable cycling of LMBs under complex operating conditions.

## Conclusions

In summary, we designed a series of solvent molecules with varying solvation effects, using DME and DOL as the primary frameworks, and systematically explored the relationship between weak solvation abilities and the electrochemical performance of the electrolytes. Our findings indicate that the electrochemical behavior of LMBs is not solely governed by the solvation structure of the electrolyte but is also influenced by the inherent reactivity of the solvent. Specifically, an electrolyte formulated with 2-MeO DOL, which exhibits both weak solvation ability and low reactivity, demonstrated the best cycling stability in LMBs. Electrochemical measurements showed that the 2-MeO DOL-based electrolyte supported stable cycling of the Li–Cu half-cell for over 250 cycles, maintaining an average CE greater than 99%. Moreover, it facilitated reliable cycling of the Li-NCM811 full cell even under challenging conditions (50 μm Li, 14.0 mg cm^−2^). This work offers essential theoretical guidance for the design and high-throughput screening of next-generation electrolytes for LMBs.^46,47^

## Data availability

Data are available from the authors on reasonable request.

## Author contributions

Z. J. and L. L. conceived and designed this work. L. L., K. R. and W. X. carried out the synthesis, electrochemical measurements and computational calculations. Z. J., L. L., Q. Y., S. W., H.-W. L., M. Y. and Y. L. participated in the analysis of the data. All authors discussed and revised the manuscript.

## Conflicts of interest

There are no conflicts to declare.

## Supplementary Material

SC-016-D5SC01495F-s001
