# Supplementary material for: Do weaker solvation effects mean better performance of electrolytes for lithium metal batteries?
Source: Chem Sci. 2025 Mar 31;16(18):7981–8. doi: 10.1039/d5sc01495f (PMC11973923; doi:10.1039/d5sc01495f)
Supplement: SC-016-D5SC01495F-s001 [file SC-016-D5SC01495F-s001.pdf]

Supplementary Information (SI) for Chemical Science.
This journal is © The Royal Society of Chemistry 2025

Electronic Supplementary Information

Do weaker solvation effects mean better performance of electrolytes for lithium metal batteries?

Liang Li[a], Kaixiang Ren[a], Wenjun Xie[a], Qi Yua, Shilin Wu[a], Hai-Wen Li[c], Meng Yao[d], Zhipeng Jiang[*a,b] and Yongtao Li[*a,b]

[a] *School of Materials Science and Engineering, Anhui University of Technology, Maanshan 243002, China.*

[b] *Key Laboratory of Efficient Conversion and Solid-state Storage of Hydrogen & Electricity of Anhui Province, Maanshan 243002, China.*

[c] *School of Advanced Energy, Sun Yat-sen University, Shenzhen 518107, China.*

[d] *College of Materials Science and Engineering, Sichuan University, Sichuan 610065 Chengdu, People's Republic of China.*

E-mail: jzp1994@ahut.edu.cn, liyongtao@ahut.edu.cn

1. Experimental Section

Material preparation

Lithium bis(fluorosulfonyl)imide (LiFSI, 99.9%), Lithium hexafluorophosphate ($LiPF_6$ 99.9%), diethyl carbonate (DEC, 99.9%), ethylene carbonate (EC, 99.9%), 1,2-dimethoxyethane (DME, 99.9%), and 1,3-dioxolane (DOL, 99.9%) were sourced from Dodo Chem. 1,2-Dimethoxypropane (DMP, 98.0%), polyvinylidene fluoride (PVDF), and N-methylpyrrolidone (NMP) were sourced from Aladdin. 1,1,2-Trimethoxyethane (TME, 98.0%), 2-methyl-1,3-dioxolane (2-Me DOL, 98.0%), and 2-methoxy-1,3-dioxolane (2-MeO DOL, 98.0%) were sourced from Adamas-Bate. Li foil (diameter 14 mm, thickness 50 μm or 400 μm) was supplied by China Energy Lithium Co., Ltd. Cu foil (16 μm) was purchased from Shenzhen Jingliang Copper Industry Co., Ltd. Al foil (14 μm), carbon-coated Al foil (15+2 μm), $LiNi_{0.8}Mn_{0.1}Co_{0.1}O_2$ (NCM811) powder, superconducting carbon black conductive agent (Super P), and double-sided ceramic separators (12+2+2 μm) were provided by Guangdong Canrd New Energy Technology Co., Ltd. Molecular sieve was added to DMP, TME, 2-Me DOL, and 2-MeO DOL before use. Other materials were not purified.

Electrolyte preparation

All electrolyte compositions contained 1.5 M LiFSI in DME, 1.5 M LiFSI in DMP, 1.5 M LiFSI in TME, 1.5 M LiFSI in DOL, 1.5 M LiFSI in 2-Me DOL, and 1.5 M LiFSI in 2-MeO DOL. Conventional carbonate electrolytes consisted of 1 M $LiPF_6$ in EC/DEC (v/v= 1: 1). All electrolytes were prepared in a glovebox under an argon atmosphere (O_2< 0.1 ppm, H_2O< 0.1 ppm).

Characterizations

Raman spectra were acquired using a 785 nm laser with a HORIBA LabRAM HR Evolution spectrometer. Lithium-ion conductivities were assessed using a DDS-307A conductivity tester. X-ray photoelectron spectroscopy (XPS) measurements were conducted using a Thermo Fisher Scientific K-Alpha instrument. The morphology of lithium deposition was investigated using a Zeiss Sigma 300 scanning electron microscope (SEM). ^{7}Li Nuclear magnetic resonance (NMR) spectroscopy was performed on a Bruker 600 MHz instrument with 1 M LiCl in D_2O as the internal

standard. Inductively coupled plasma mass spectrometry (ICP-MS) was measured with an Agilent 7800 (MS).

Electrochemical measurements

Low-loading cathodes were prepared by scraping a mixture of NCM811, Super P, and PVDF in a ratio of 8: 1: 1, followed by the addition of an NMP solution. The ratio of NCM811, Super P, and PVDF used in the high-loading cathodes was 9: 0.5: 0.5, with other conditions the same as those of the low-loading cathodes. The resulting slurry was stirred using a high-speed vibrating ball mill before being coated onto carbon-coated Al foil of varying thickness using a coater. The coated electrode material was subsequently dried at 80 °C for 10 h in a conventional drying oven. Li-Cu half cells, Li-NCM811 cells, and Li-Al cells were assembled using 2032-type coin cells within a glovebox filled with argon gas (H_2O< 0.1 ppm, O_2< 0.1 ppm). The Li-Cu half cells were assembled using Li foil (ϕ14 mm, 400 μm) and Cu foil (ϕ16 mm, 16 μm). Li-NCM811 cells (3.0-4.4 V) were constructed using Li foil (ϕ14 mm, 400 μm) and low-loading NCM811 cathodes (ϕ8 mm, 2.0 mg cm^{-2}) with the studied electrolytes for rate and cycling tests. The Li-NCM811 full cell (3.0-4.4 V) was prepared by combining Li foil (ϕ14 mm, 50 μm) with high-loading NCM811 cathodes (ϕ8 mm, 14 mg cm^{-2}) using a 2-MeO DOL electrolyte for cycling tests. The Li-Al cell configuration involved Li foil (ϕ14 mm, 400 μm) and Al foil (ϕ16 mm, 14 μm). The coin cells for all configurations employed a double-sided ceramic separator with an electrolyte volume of 40 μL. The electrochemical tests were conducted using the LAND test system at 25 °C or -20 °C. Linear sweep voltammetry (LSV) measurements were performed on the Li-Al cell using the CHI660E electrochemical workstation at a scan rate of 1 mV s^{-1}. Tafel plot analysis was conducted on the Li-Li symmetric cell using the CHI660E electrochemical workstation, covering a voltage range of -0.2 V to 0.2 V with a sweep rate of 1 mV s^{-1}. Li-NCM811 cell impedance was tested on the CHI660E electrochemical workstation with a voltage amplitude of 5 mV in the frequency range of 100 kHz to 0.1 Hz. The differential pulse voltammetry (DPV) is used to test Li-Cu batteries in CHI660E electrochemical work and DPV measurements were conducted with the

parameters: an initial potential step of 5 mV, pulse amplitude of 50 mV, pulse duration of 1 s, and pulse interval of 2 s.

Theoretical calculations

All calculations were performed using the DMol3 code with the generalized gradient approximation (GGA) method, employing a double numerical plus polarization (DNP) basis set for all elements involved based on density functional theory (DFT). During structural optimization, the convergence criteria for energy, force, and maximum displacement were set to 1×10^{-5} Ha, 0.002 Ha/Å, and 5×10^{-3} Å, respectively.

The molecular dynamics (MD) simulations were performed using the GROMACS 2023.02 software package, with all molecules described by the OPLS-AA force field. Initial configurations for all simulated systems were generated using the Packmol software package to uniformly mix the components. The systems first underwent energy minimization for 3,000 steps using the steepest descent algorithm to eliminate unreasonable atomic overlaps. Subsequently, equilibration was conducted at 298.15 K in both NVT and NPT ensembles, each using a 1.0 fs integration time step for 100 ps. Production simulations were then carried out for 20 ns under the NPT ensemble. Periodic boundary conditions were applied in all three directions, with a 2.0 fs time step during MD simulations. Temperature was maintained at 298.15 K using the V-rescale thermostat (coupling time: 0.5 ps), while pressure was controlled at 1 bar using the Parrinello-Rahman barostat (coupling time: 2.0 ps). All hydrogen-containing bonds (C–H, O–H) were constrained using the LINCS algorithm. Long-range electrostatic interactions were treated using the Particle Mesh Ewald (PME) method, while short-range electrostatic and van der Waals (vdW) interactions were calculated with a 1.2 nm cutoff. Trajectory analysis and post-processing were performed using the GROMACS software package.

2. Supplementary Figures

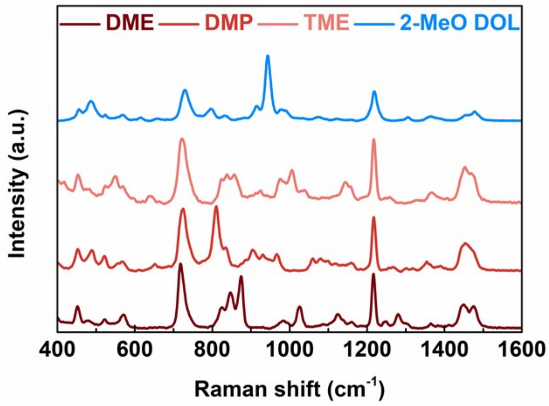

Fig. S1 Raman spectra comparison of different ether electrolytes.

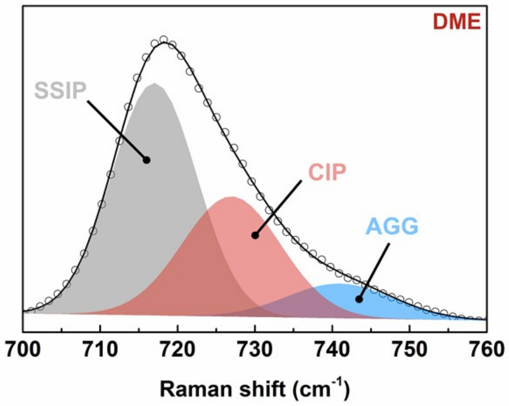

Fig. S2 Deconvoluted peaks of Raman spectra of 1.5 M LiFSI in DME.

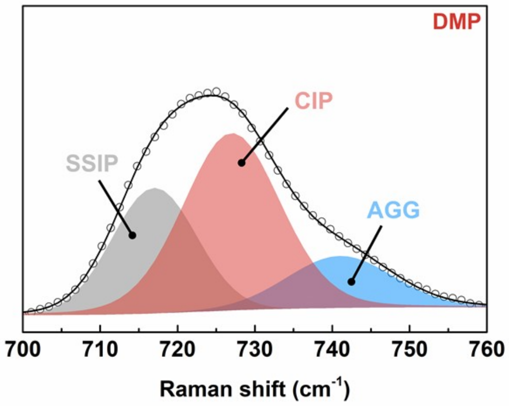

Fig. S3 Deconvoluted peaks of Raman spectra of 1.5 M LiFSI in DMP.

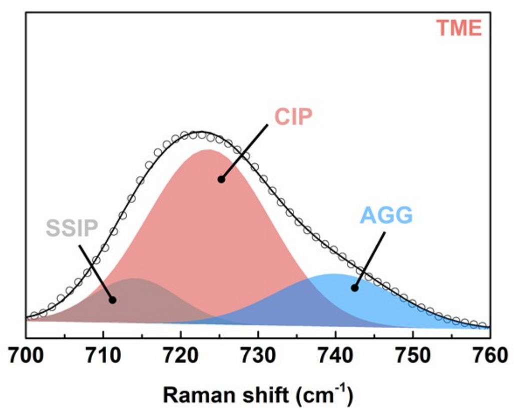

Fig. S4 Deconvoluted peaks of Raman spectra of 1.5 M LiFSI in TME.

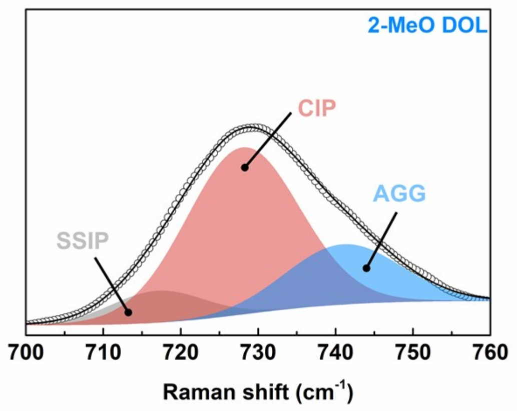

Fig. S5 Deconvoluted peaks of Raman spectra of 1.5 M LiFSI in 2-MeO DOL.

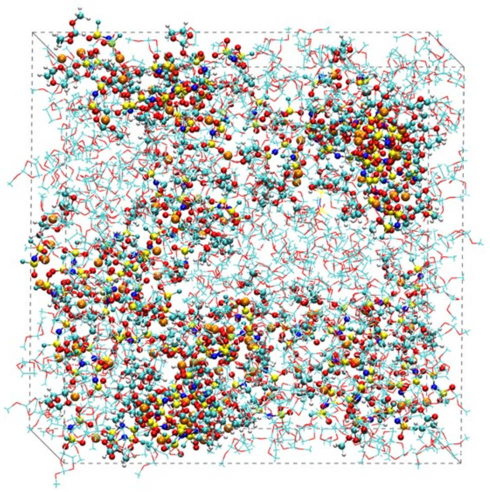

Fig. S6 MD simulation snapshots cell of 1.5 M LiFSI in 2-MeO DOL.

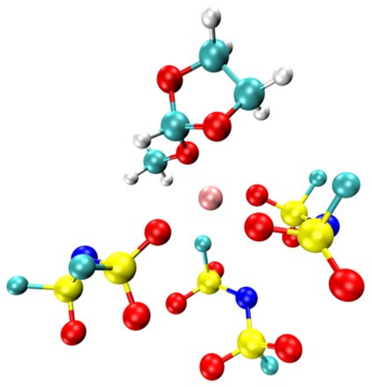

Fig. S7 The partial Li solvation structure of 1.5 M LiFSI in 2-MeO DOL.

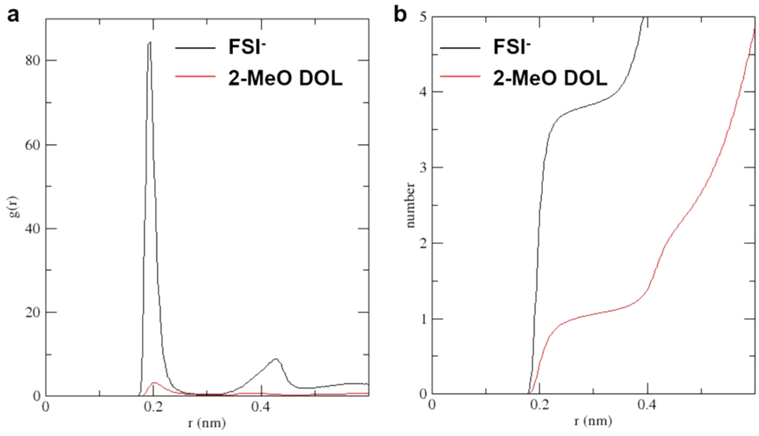

Fig. S8 (a) The radial distribution functions of 1.5 M LiFSI in 2-MeO DOL. (b) The coordination number near Li^+.

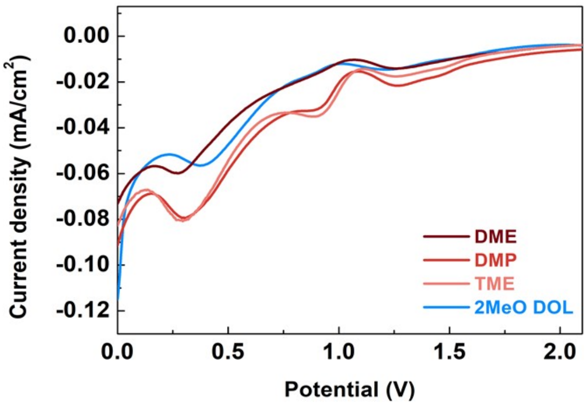

Fig. S9 DPVs of Li-Cu cell using different electrolytes.

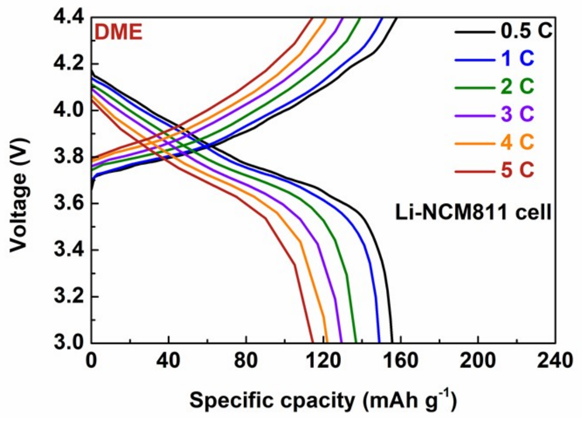

Fig. S10 Typical voltage profiles of the Li-NCM811 cell using 1.5 M LiFSI in DME for rate test.

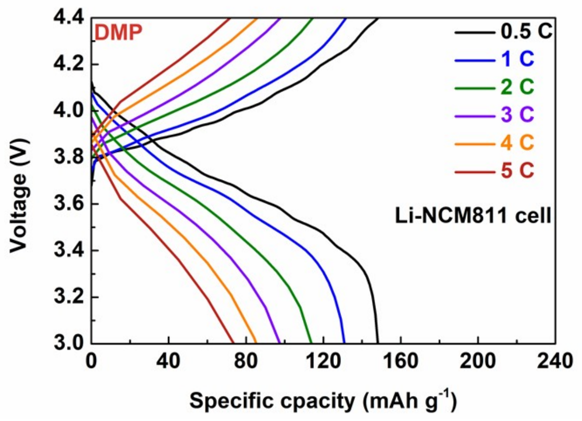

Fig. S11 Typical voltage profiles of the Li-NCM811 cell using 1.5 M LiFSI in DMP for rate test.

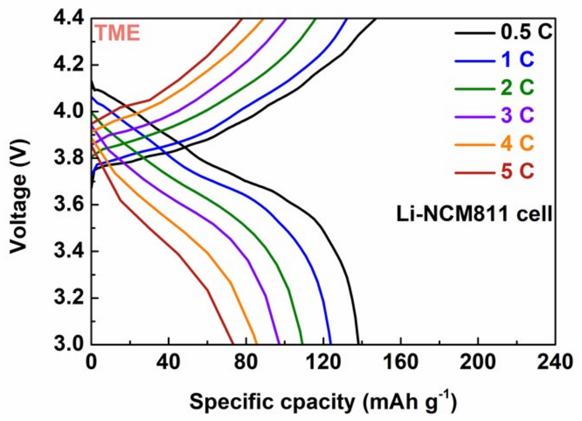

Fig. S12 Typical voltage profiles of the Li-NCM811 cell using 1.5 M LiFSI in TME for rate test.

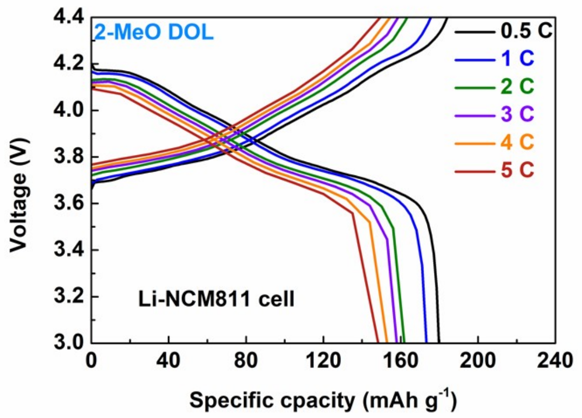

Fig. S13 Typical voltage profiles of the Li-NCM811 cell using 1.5 M LiFSI in 2-MeO DOL for rate test.

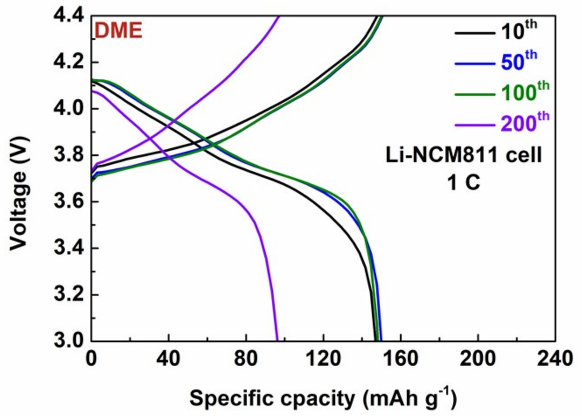

Fig. S14 Typical voltage profiles of the Li-NCM811 cell using 1.5 M LiFSI in DME for long-term cycling test at 1 C..

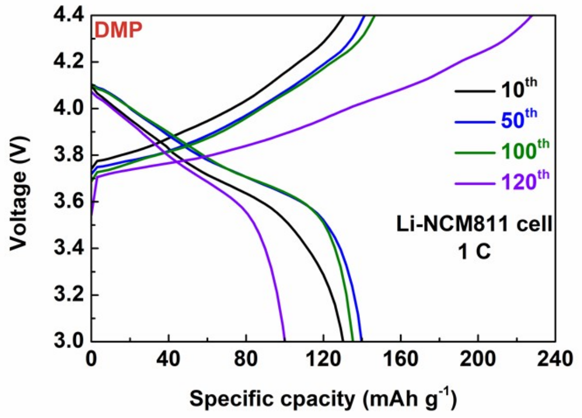

Fig. S15 Typical voltage profiles of the Li-NCM811 cell using 1.5 M LiFSI in DMP for long-term cycling test at 1 C.

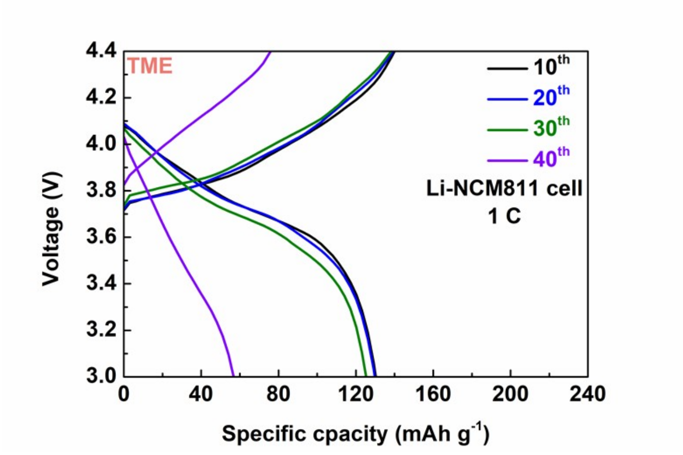

Fig. S16 Typical voltage profiles of the Li-NCM811 cell using 1.5 M LiFSI in TME for long-term cycling test at 1 C.

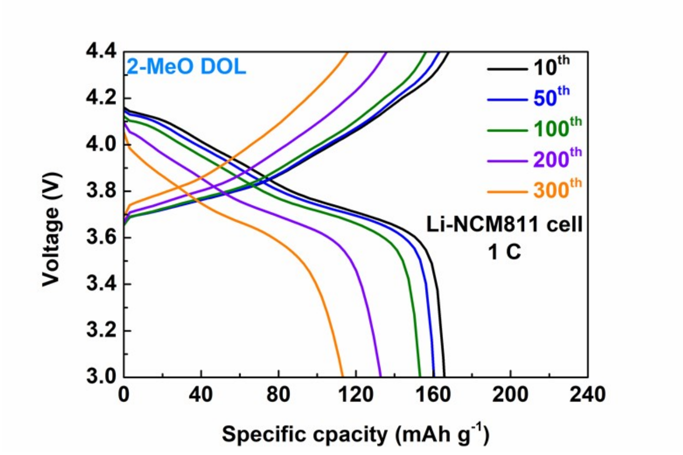

Fig. S17 Typical voltage profiles of the Li-NCM811 cell using 1.5 M LiFSI in 2-MeO DOL for long-term cycling test at 1 C.

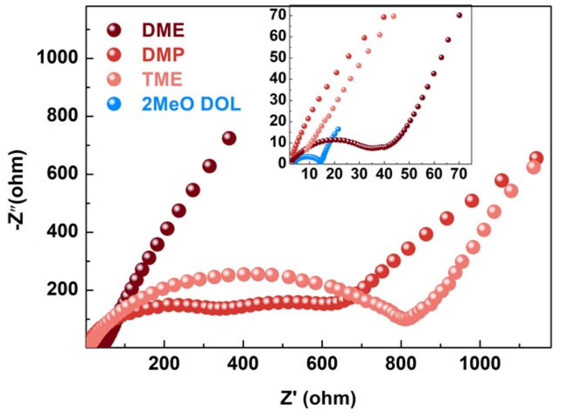

Fig. S18 EIS of Li-NCM811 cells using different electrolyte after initial cycle.

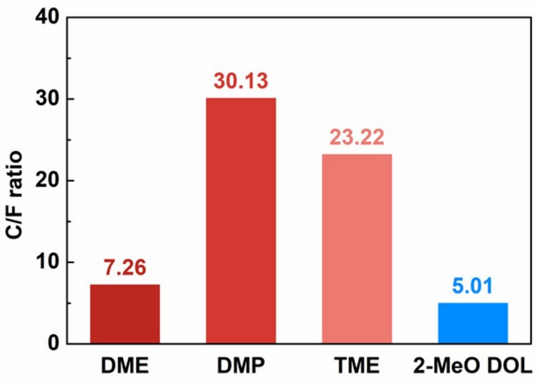

Fig. S19 Comparison of C/F ratios in SEI for deposited Li using different ether electrolytes obtained from XPS.

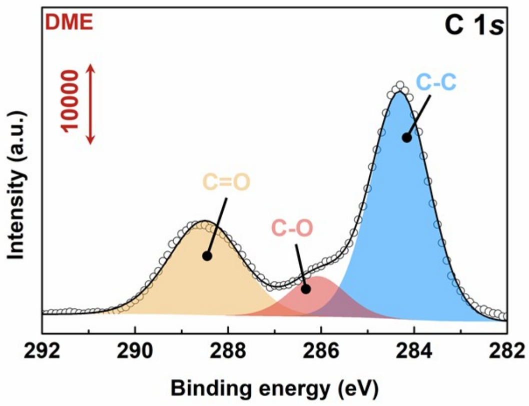

Fig. S20 XPS C 1*s* spectra of deposited Li using 1.5 M LiFSI in DME

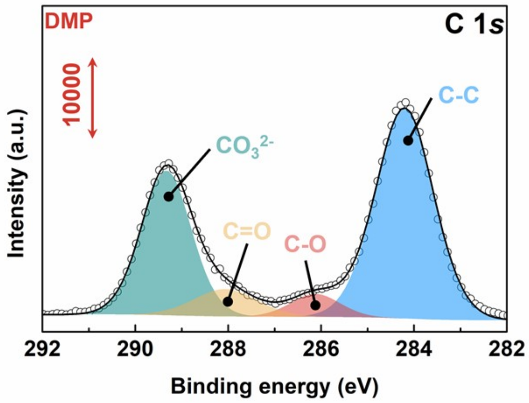

Fig. S21 XPS C 1*s* spectra of deposited Li using 1.5 M LiFSI in DMP.

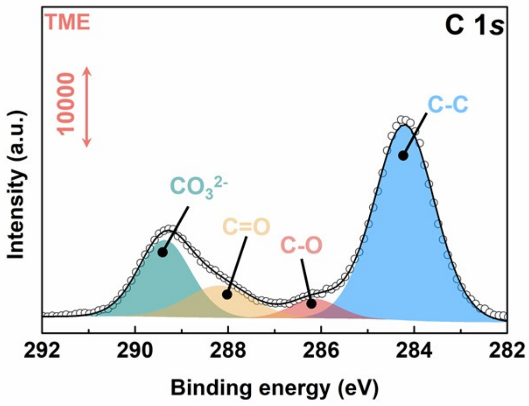

Fig. S22 XPS C 1*s* spectra of deposited Li using 1.5 M LiFSI in TME.

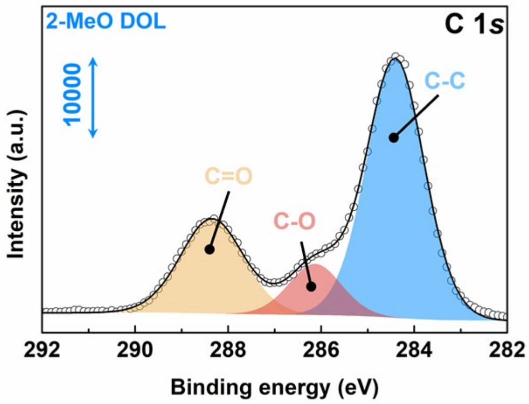

Fig. S23 XPS C 1*s* spectra of deposited Li using 1.5 M LiFSI in 2-MeO DOL.

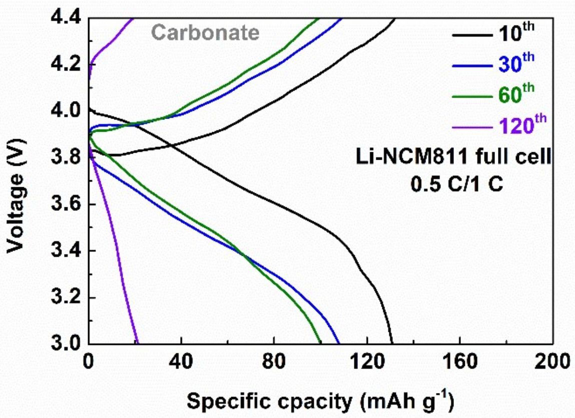

Fig. S24 Typical voltage profiles of the Li-NCM811 full cell using carbonate electrolyte for long-term cycling test at 0.5 C/1 C.

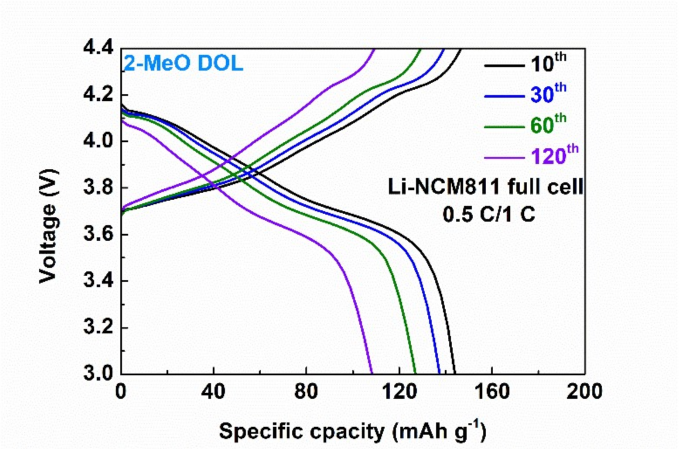

Fig. S25 Typical voltage profiles of the Li-NCM811 full cell using 1.5 M LiFSI in 2-MeO DOL for long-term cycling test at 0.5 C/1 C.

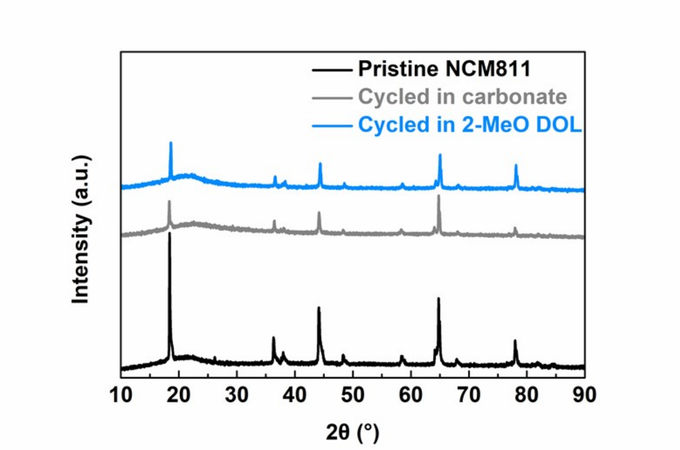

Fig. S26 XRD patterns of pristine and cycled NCM811 cathodes in Li-NCM811 full cell using different electrolytes.

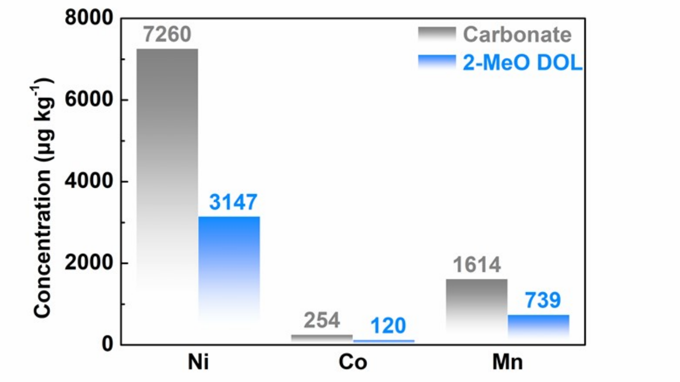

Fig. S27 The contents of Ni, Mn, and Co deposited on cycled metallic Li in Li-NCM811 full cell were tested by ICP-MS.

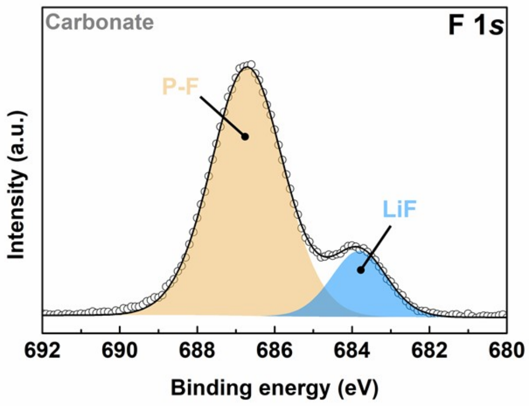

Fig. S28 XPS C 1*s* spectra of cycled NCM811 cathode using carbonate electrolyte.

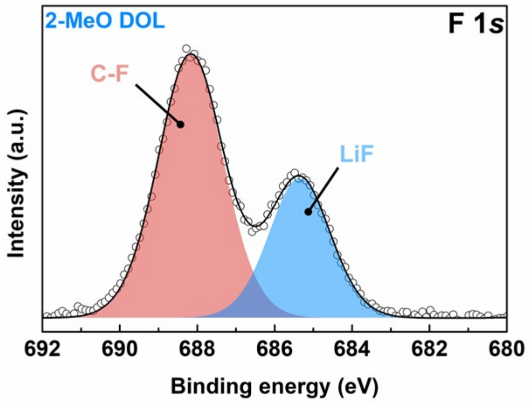

Fig. S29 XPS C 1*s* spectra of cycled NCM811 cathode using 1.5 M LiFSI in 2-MeO DOL.

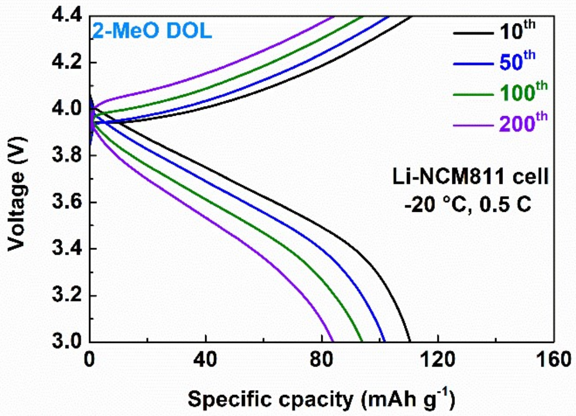

Fig. S30 Typical voltage profiles of the Li-NCM811 cell using 1.5 M LiFSI in 2-MeO DOL for low-temperature long-term cycling test at -20 °C/0.5 C.
